# Acute Lymphoblastic Leukemia Characterized by Rare *BCR*::*FGFR1* Translocation: A Case Report With Literature Review

**DOI:** 10.1155/crh/8892036

**Published:** 2025-10-23

**Authors:** Maximilian Al-Bazaz, Anika Forstreuter, Ibrahim Hammada, Jurek Hille, Jan Nicolai Wagner, Jochim Reinert, Janine Wehrhahn, Carsten Bokemeyer, Walter Fiedler

**Affiliations:** ^1^Department of Oncology, Hematology and Bone Marrow Transplantation with Division of Pneumology, University Medical Center Hamburg-Eppendorf, Hamburg, Germany; ^2^Department of Cytogenetics, Medical Care Center for Human Genetics and Molecular Pathology GmbH, Rostock, Germany

**Keywords:** 8p11 myeloproliferative syndrome, acute lymphoblastic leukemia, allogeneic stem cell transplantation, BCR::FGFR1 translocation, *breakpoint cluster region*(*BCR*) and *fibroblast growth factor receptor 1*(*FGFR1*), case report

## Abstract

**Case:**

We present the case of a 60-year-old male patient with a common B-cell acute lymphoblastic leukemia (ALL) who carried the rare t(8; 22)(p11; q11) *BCR*::*FGFR1* chromosomal translocation.

**Background/Objectives:**

The presence of the t(8; 22)(p11; q11) *BCR*::*FGFR1* translocation, identified by cytogenetics including Fluorescence In Situ Hybridization (FISH) is known for its association with aggressive disease. Given the dismal prognosis, an early search for a stem cell donor was initiated.

**Methods:**

The patient was treated according to the German Multicenter ALL (GMALL) Study Group consensus recommendations. The disease was refractory to the first cycle of induction chemotherapy. However, after the second induction, cytological remission was achieved. Nevertheless, minimal residual disease (MRD) positivity persisted (IGH rearrangement detected by PCR) after the first consolidation therapy, giving indication for a stem cell transplantation (SCT).

**Results:**

Thirty days post-transplant, no MRD was detected, and complete chimerism was measured for the months following transplantation. However, the patient died in the context of severe graft-versus-host disease and infectious complications 6 months after the SCT.

**Conclusions:**

This case highlights the importance of detailed molecular analysis in the initial diagnostics of ALL. Identification of specific chromosomal translocations can provide critical insights for risk assessment and aid decision-making in intensify therapeutic approaches.

**Trial Registration:**

ClinicalTrials.gov identifier: NCT03011372, NCT04659616

## 1. Introduction

Acute lymphoblastic leukemia (ALL) is a hematologic malignancy comprising various subtypes, each defined by characteristic cytogenetic and molecular markers according to the WHO (2022) and the ICC (2022) [1–4].

A notable example of the pathogenic, prognostic, and predictive significance of a single aberration is the t(9; 22)(q34; q11.2) translocation, which leads to the formation of the Philadelphia chromosome (Ph) and the resultant *BCR::ABL1* fusion gene, a well-established marker in ALL [5, 6]. In the presented case report, a rare activating t(8; 22)(p11; q11) *BCR*::*FGFR1* chromosomal translocation was identified.

Fibroblast growth factor receptor 1 (FGFR1), a member of the FGFR receptor family, regulates cellular processes such as proliferation, differentiation, migration, survival, and morphology. The *BCR*::*FGFR1* fusion results in constitutive activation of FGFR1, driving oncogenesis by persistently stimulating key intracellular pathways, including RAS‍–RAF–MEK–MAPK, which promotes uncontrolled cell division and differentiation, and PI3K–AKT–mTOR, which inhibits programmed cell death [7–10].

Several case reports and studies have highlighted the critical role of FGFR1 in the development of myeloid and lymphoid neoplasms [11–13]. The translocation has been reported in a variety of hematologic malignancies, including chronic myeloid leukemia (CML), myeloproliferative neoplasms (MPN), ALL, and acute myeloid leukemia (AML). Despite increasing recognition, treatment remains challenging, and standardized therapeutic protocols have yet to be established [14–19].

## 2. Case Presentation

A 60-year-old male patient presented with a significant decline in performance status and petechiae. B symptoms, bleeding, and signs of infection were not present.

His past medical history included colon cancer more than 10 years prior, successfully treated with surgery and adjuvant chemotherapy with capecitabine. Additional pre-existing conditions included arterial hypertension and diabetes mellitus. The patient was an ex-smoker with a cumulative history of approximately 40 pack-years, and occasional alcohol consumption was reported.

### 2.1. Clinical Findings

The 60-year-old patient presented in stable overall condition with normal nutritional status. Physical examination revealed petechiae on the lower legs and was, otherwise, unremarkable.

### 2.2. Diagnostic Assessment

Computer tomography revealed the presence of splenomegaly and a suspicious lymph node in the porta hepatis region; otherwise, no lymphadenopathy was detected.

**Figure 1:**
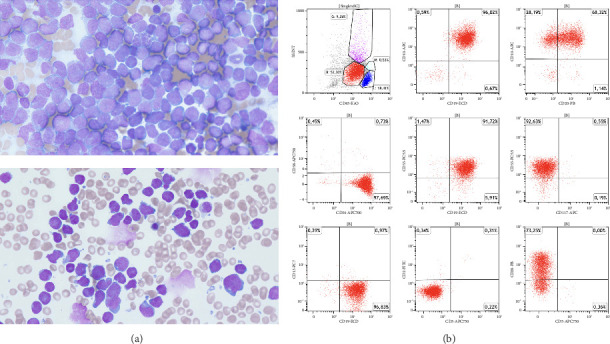
(a) Cytomorphological examination of bone marrow smear (top) and peripheral blood smear (bottom) at the time of initial diagnosis. The peripheral blood contained 33% blasts, while the bone marrow aspirate revealed 95% blasts in a hypercellular bone marrow. The blasts appear as medium-sized cells with a round to oval nucleus, a fine chromatin pattern, and prominent nucleoli. They exhibit a narrow rim of basophilic cytoplasm, characteristic of immature precursor cells, indicative of leukemia. (b) Exemplary key findings from FACS analysis of the bone marrow, demonstrating a common B-ALL phenotype with aberrant expression of CD33. Leukocytes are depicted, with blasts highlighted in red after gating based on the CD45+/SSC plot (as shown).

His complete blood count showed leukocytosis of 70,300/μL with neutrophils at 6970/μL, lymphocytes at 57,620/μL, and monocytes at 17,113/μL. Anemia was evident, with a hemoglobin level of 6.5 g/dL, and thrombocytopenia was confirmed with a platelet count of 14,000/μL. Eosinophilia was not observed, with an eosinophil count of 170/μL. Cytomorphological examination of the peripheral blood smear identified 33% of the cells as blasts. Bone marrow aspiration for cytology revealed 95% blasts and bone marrow biopsy showed a high cellularity with a 90% infiltration of B-cell ALL. The blast population showed expression of cyCD79a, CD10, CD19, CD34, and TdT, consistent with common B-ALL phenotype. Notably, there was an aberrant expression of myeloid marker CD33 (Figure 1).

**Figure 2:**
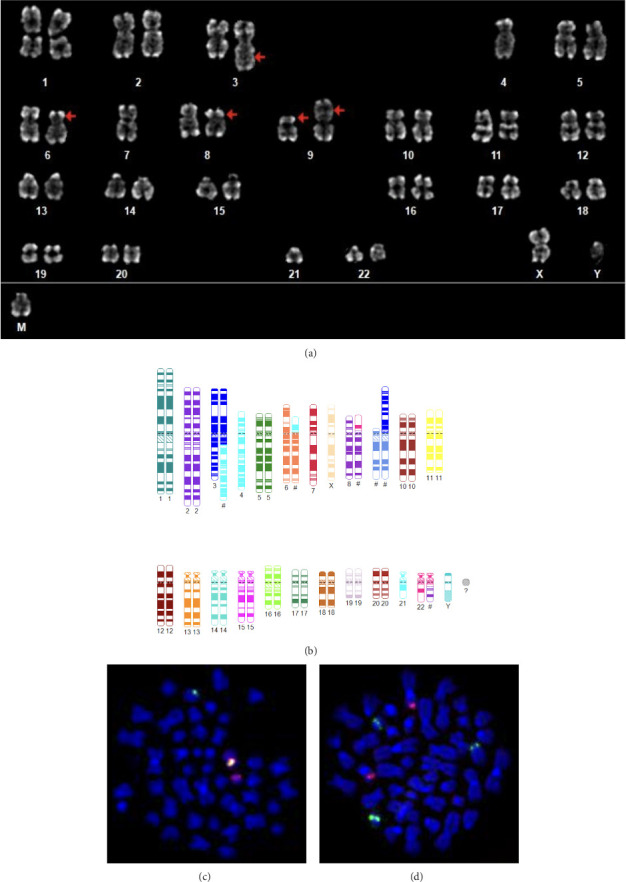
The patient's complex karyotype. (a) Detailed karyotype illustrating the BCR::FGFR1 translocation alongside additional structural anomalies affecting chromosomes 3, 4, 6, 7, 9, and 21. Red arrows denote translocations, emphasizing the chromosomal rearrangements. The complete karyotype is as follows: 44,XY,der(3)t(3; ?4)(q13; q?11),-4,der(6)? t(6; 21)(p11; q11),-7,t(8; 22)(p11.2; q11.2),del(9)(p12p2?3),der(9)t(3; 9)(q11; p11),-21,+mar[cp12]. (b) Schematic representation of the karyogram using CyDAS [20], illustrating the structural chromosomal abnormalities in a simplified visual format, with permission to use from Medical Care Center for Human Genetics and Molecular Pathology GmbH, Rostock, Germany. (c) FISH analysis on metaphase chromosomes using an FGFR1 break-apart probe with one green-orange fusion signal on the normal chromosome 8 and one separated signal: the proximal green signal corresponding to FGFR1 remained on the aberrant homolog of chromosome 8, while the distal orange signal was translocated to the aberrant chromosome 22. (d) FISH analysis on metaphase chromosomes using a BCR::ABL1 dual-color, dual-fusion translocation probe revealed one intact green signal for the BCR locus on the normal chromosome 22 and two split green signals: one remaining on the aberrant homolog of chromosome 22 and one translocated to the aberrant chromosome 8. The orange-labeled ABL1 locus was observed on two aberrant chromosome 9 homologs.

Initial cytogenetic and Fluorescence In Situ Hybridization (FISH) analysis (Figure 2) revealed a complex karyotype with indirect detection of the *BCR*::*FGFR1* translocation and *CDKN2A* deletion. Furthermore, structural and numerical alterations were observed in chromosomes 3, 4, 6, 7, and 21.

The complete karyotype was as follows:

  44,XY,der(3)t(3; ?4)(q13; q?11),-4,der(6)? t(6; 21)(p11; q11),-7,t(8; 22)(p11.2; q11.2), del(9)(p12p2?3),der(9)t(3; 9)(q11; p11),-21,+mar[cp12].
  Detection of the *BCR*::*ABL1* fusion gene by RT-PCR was negative.

At the time of diagnosis, a molecular minimal residual disease (MRD) marker (*IGH* rearrangement) was established for follow-up assessments using real-time quantitative polymerase chain reaction (RQ-PCR). This technique is highly sensitive, with a detection level of 1E-05. PCR primers and protocols are described in the Supporting Information (available here).

### 2.3. Treatment and Course

The patient was treated according to the German Multicenter ALL (GMALL) protocol for elderly patients over 55 years old (Version 3) [21]. The GMALL protocol is a pediatric-inspired protocol designed for adult patients with ALL, incorporating risk- and MRD-adapted chemotherapy and stem cell transplantation (SCT) regimens, central nervous system prophylaxis, and standardized follow-up procedures to improve patient outcomes. The patient started with prephase therapy using dexamethasone, cyclophosphamide, and intrathecal methotrexate, followed by remission–induction cycle 1 with rituximab, vincristine, idarubicin, and dexamethasone. Central nervous system prophylaxis was administered with intrathecal triple therapy comprising cytarabine, methotrexate, and dexamethasone.

Bone marrow aspirate after first part of induction therapy revealed significant persistence of blasts, with 65% still present in the marrow. Phenotypic assessment revealed an unchanged phenotype, still consistent with common B-ALL with aberrant CD33 expression. Therapy proceeded immediately to the second part of induction, which included rituximab, cyclophosphamide, and cytarabine, as well as continued central nervous system prophylaxis with intrathecal triple therapies.

Bone marrow aspiration after the completion of induction cycle showed cytomorphological remission; however, molecular analysis for MRD detected 0.2% residual infiltration of leukemic blasts.

According to the protocol, the patient then underwent consolidation therapy including high-dose methotrexate, peg-asparaginase, and rituximab. Despite this, MRD levels persisted at 0.03%.

The therapeutic course was complex but proceeded without major interruptions or dose reductions. Complications of the induction and consolidation chemotherapy included grade 3 anemia and grade 4 thrombocytopenia, both requiring transfusion of blood products. The thrombocytopenia led to a subdural hematoma and a generalized seizure; however, neurosurgical intervention was not required. Further complications included chemotherapy-associated hyperbilirubinemia, steroid-induced hyperglycemia, peg-asparaginase-induced hypertriglyceridemia, elevated lipase levels, antithrombin III deficiency, and supraventricular tachycardia, which was successfully managed with metoprolol and electrolyte replacement.

At the time of treatment, there was no EMA approval for blinatumomab below an MRD level of 0.1%. Consequently, the patient underwent allogeneic hematopoietic SCT from a matched unrelated donor. On day 30 after transplantation, the molecular MRD marker was negative in the bone marrow. Repeated cytogenetic analysis revealed no evidence of the *BCR*::*FGFR1* translocation. The case was discussed extensively by the tumor board; due to a lack of data, there was no indication for targeted therapy against *BCR*::*FGFR1*. Furthermore, since MRD negativity was achieved through conventional treatment, the use of tyrosine kinase inhibitors (TKIs) was not justified.

The patient was hospitalized 6 weeks post-transplantation due to angioedema from an allergic reaction. A skin rash was also confirmed as GvHD.

Three weeks later, the patient was readmitted with suspected encephalitis though the cause remained unclear. Neurological symptoms eventually improved, but skin desquamation worsened again. Subsequently, bloodstream infections with *Enterococcus faecium* and *Candida glabrata* were diagnosed and treated appropriately. A second skin biopsy revealed a medical-toxic reaction rather than GvHD, necessitating adjustments in treatment.

**Figure 3:**
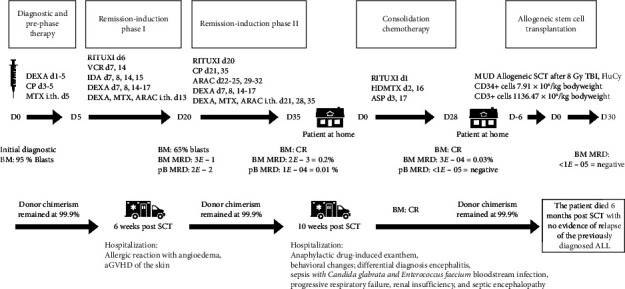
Timeline of hematological procedure. Therapy in accordance with the GMALL protocol for elderly patients over 55 years old (Version 3); d1 = Day 1; DEXA = dexamethasone; CP = cyclophosphamide; MTX = methotrexate; RITUXI = rituximab; VCR = vincristine; IDA = idarubicin; ARAC = cytarabine; HDMTX = high-dose methotrexate; ASP = peg-asparaginase; TBI = total body irradiation; FluCy = conditioning regimen with fludarabine and cyclophosphamide; BM = bone marrow; CR = complete remission; MRD = minimal residual disease (with sensitivity of 1*E *− 05 = equivalent to 0.001% of the nucleated cells); aGVHD = acute graft-versus-host disease.

The patient was transferred back to the hematology ward but later developed pneumonia and septic shock, requiring ICU care. Despite initial stabilization, a second septic event led to worsening health, intubation, and ultimately multiorgan failure. The patient died 6 months after transplantation, with no signs of relapse, and complete remission was confirmed 2 months before death. Donor chimerism in the peripheral blood was still 99.9% days before death. A timeline of the hematological procedure is shown in Figure 3.

## 3. Discussion

Overall, the *BCR*::*FGFR1* translocation is extremely rare, which prompted us to conduct a literature review upon diagnosis, revealing descriptions of rapidly progressive and challenging-to-treat leukemia. The GMALL treatment protocol for acute lymphatic leukemia was followed, including an MRD-based approach regarding consolidative hematopoietic SCT. However, upon admission, we initiated HLA typing and began the search for a suitable donor.

Of all patients with ALL, only a minority of patients undergoes allogeneic SCT. Despite this, the majority of patients can be cured without SCT [22]. However, for ALL patients who remain MRD positive and do not undergo allogeneic SCT, the recurrence probability remains very high and relapse occurs after a median of 7.6 months [23].

Despite achieving hematological remission post-transplantation, our patient ultimately died from nonrelapse mortality, highlighting the risks associated with allogeneic hematopoietic SCT. Nonrelapse mortality remains a significant obstacle to its success, with complications such as graft-versus-host disease, infections, and organ dysfunction contributing to mortality rates independent of disease relapse. Even in the absence of leukemia relapse, the post-transplantation period is fraught with life-threatening complications that require proactive and coordinated care efforts.

The *BCR*::*FGFR1* translocation found in hematological malignancies has previously been described in a subset of MPNs, which were historically designated as “8p11 myeloproliferative syndromes,” a rare and aggressive group of disorders characterized by genetic rearrangements involving *FGFR1* on chromosome 8p11–12.

To date, slightly over 100 cases have been documented, involving one of 17 different fusion genes including the BCR gene located on chromosome 22 [13, 24, 25]. The disease with this translocation is now classified as myeloid/lymphoid neoplasm with eosinophilia and tyrosine kinase gene fusion in the 5^th^ edition of the WHO and the International Consensus Classification of Myeloid Neoplasms and Acute Leukemias 2022 [3, 4].

Few similar cases have been reported in the literature; this represents the 34th published case of a myeloid/lymphoid neoplasm with t(8; 22)/*BCR*::*FGFR1* rearrangement. Approximately one third of the reported cases presented as B-ALL, while the remaining cases were diagnosed as MPN or AML. All reported cases are shown in Table 1.

In certain cases, signs with diagnostic features of an MPN were noticeable either at initial diagnosis or manifested during the disease's chronic stage after undergoing chemotherapy for B-ALL, linked to the t(8; 22)(p11.2; q11.2)/*BCR*::*FGFR1* rearrangement.

As described in the literature, the *BCR*::*FGFR1* translocation has been observed in cases with mixed phenotype leukemia. Interestingly, the presented case of common B-ALL also showed aberrant expression of the myeloid marker CD33. These findings may support the hypothesis that the cell of origin in 8p11 myeloproliferative syndrome is a pluripotent stem cell [34, 36].

Furthermore, Wang et al. (2016) reported a case of B-ALL with an initial karyotype 47, XX,t (8, 22) (p11.2; q11.2) + der (22)t (8, 22) in molecular remission (negative Flow-MRD) after chemotherapy, although bone marrow cytogenetics still showed the persistent presence of t (8, 22) (p11.2; q11.2), even in myeloid cells. Notably, the additional derivative chromosome der(22)t(8; 22), initially identified, was not detectable post-therapy. They concluded that acquisition of an additional cytogenetic abnormality played a critical role in progression to a lymphoblastic phase [17]. Similar findings were reported by Haslam et al. A patient with an initially complex karyotype received therapy according to UKALL 2003 Protocol. Remission control showed MPN morphology with an absence of blasts; however, cytogenetics still confirmed the presence of t(8; 22) as the sole aberration [35].

It is assumed that neoplastic cells with only t(8; 22) are more likely to present as a chronic myeloproliferative neoplasm, while acquiring additional cytogenetic abnormalities is associated with progress to acute leukemia [17].

### 3.1. Therapeutic Strategies

A literature review conducted on the treatment of leukemia with the *BCR*::*FGFR1* translocation underscores the importance and effectiveness of allogeneic hematopoietic SCT as a primary therapy to achieve molecular remission in acute leukemia (as seen in Table 1). However, emerging evidence highlights the potential role of TKIs in the treatment regimen.

Cases reported by Dhangar et al. [14] and Wakim et al. [19] illustrate the variable efficacy of sorafenib, a multikinase inhibitor able to inhibit FGFR1, in treating leukemia with the *BCR*::*FGFR1* rearrangement [49]. Dhangar et al. demonstrated effective treatment with multikinase inhibitor sorafenib combined with azacitidine in a patient with atypical CML presenting with leukocytosis and a sole *BCR*::*FGFR1* rearrangement, after dasatinib proved ineffective [14]. However, Wakim et al. presented a case with only a transient response in a patient with B-ALL treated with the multikinase inhibitor sorafenib which also blocks FGFR1 [19, 49]. The authors attributed limited response to clonal evolution as the follow-up cytogenetics of the recurrent B-ALL revealed a more complex karyotype than that observed at initial diagnosis [19].

Similarly, a case responding to ponatinib, another TKI inhibiting FGFR1, demonstrated a partial but significant response in leukemia with this translocation [36, 50]. A patient with leukocytosis and mixed-phenotype leukemia initially received mitoxantrone, etoposide, and cytarabine (MEC) treatment, after which his leukemia rapidly progressed. Subsequently, therapy with ponatinib was initiated, and the patient responded partially with residual leukemia of 15%–20% blasts in the core biopsy (compared with 80%–90% at diagnosis). Thereafter, the patient received Hyper-CVAD Part B followed by an allogeneic hematopoietic SCT from a matched-sibling donor. Six months later, complete morphological remission was achieved, and a normal donor female karyotype was detected. However, qPCR revealed an *BCR*::*FGFR1* fusion transcript at 8.3% allele burden relative to diagnosis. Subsequently, therapy with ponatinib was resumed, and 6.5 months later, the *BCR*::*FGFR1* fusion transcript was significantly reduced to < 0.2% of the allele burden at diagnosis [36].

Laboratory studies by Chase et al. further highlight the efficacy of multitarget kinase inhibitors like dovitinib and ponatinib in targeting cells transformed by *BCR*::*FGFR1* and in primary patient-derived cells with FGFR1 translocations. These inhibitors act by reducing phosphorylation in downstream ERK and STAT5, inhibiting the growth and survival of these cell. These findings suggest that the use of these inhibitors could be a promising treatment choice for individuals diagnosed with 8p11 myeloproliferative syndrome [51, 52]. However, Barnes et al. presented conflicting results about ponatinib and dovitinib in *in vitro* drug response assays, highlighting the complexity of treating 8p11 myeloproliferative syndrome. These discrepancies suggest that the efficacy of TKIs may depend on the lineage of the leukemia (myeloid vs. lymphoid) and the specific genetic context [16].

Pemigatinib, a FGFR1-3 inhibitor, has received FDA approval for use in previously treated cases of unresectable, locally advanced, or metastatic cholangiocarcinoma characterized by FGFR2 fusion or other similar rearrangements, following the outcomes of the FIGHT-202 trial [53]. Additionally, pemigatinib has demonstrated significant antitumor efficacy in various cell lines and xenograft tumor models harboring FGFR genetic alterations [54, 55].

Clinical trial data of the FIGHT-203 trial show promising response rates with pemigatinib monotherapy in FGFR1-rearranged myeloid/lymphoid malignancies. In an evaluation of the first 33 patients (8 had the t (8, 22) translocation) for efficacy within the trial, a complete response was observed in 72.7% of the cases, with a complete cytogenetic response rate of 75.8%. Notably, many of these patients had experienced disease progression after intensive chemotherapy or hematopoietic SCT [12].

Furthermore, another clinical trial starts recruiting in Portland, USA, to determine the optimal dosage and clinical benefits of administering pemigatinib following standard induction chemotherapy in patients newly diagnosed with AML characterized by an FGFR1 translocation.

## 4. Conclusion

Detailed molecular analysis plays a critical role in the initial diagnosis of ALL. The identification of specific chromosomal translocations provides valuable insights into disease biology and informs risk assessment. This knowledge not only guides therapeutic decisions, particularly regarding treatment intensification, but also enables the development of personalized treatment strategies, ultimately improving patient outcomes and prognosis.

In summary, this case of ALL with the t(8; 22) translocation and a complex karyotype proved to be challenging to treat. It required an intensified therapeutic approach, primarily through hematopoietic SCT, to achieve molecular remission and ensure therapeutic efficacy.

Additionally, the potential utility of TKIs as a supplementary or bridging therapy in this context should be considered. The literature highlights instances where TKI therapy has been effective, but the variable responses necessitate a cautious and individualized approach. The role of TKIs, particularly in conjunction with standard treatments such as allogeneic hematopoietic SCT, offers a promising option but requires further exploration through clinical studies to more precisely define their efficacy and integration in treatment protocols.

## Figures and Tables

**Table 1:** Summary of 33 published cases of BCR::FGFR1 translocations.

Clinic	Age, sex	Cytogenetics	Treatment	Outcome	Author, date
AML	58, M	47,XY,t(8; 22)(p11.2; q11.2),+19	1. Sorafenib + 7 + 3 2. Allogeneic HSCT 3. Flag-Ida	Originally, CR with MRD negative status; relapse 5 months and death 6 months after presentation	Barnes et al., 2020 [16]
AML	74, F	46,XX,del(5)q33q35,t(8; 22)(p11; q11)	Mitoxantrone, etoposide, interferon *α*, hydroxyurea, fludarabine	Not reported	Matikas et al., 2013 [18]
AML	37, F	46,XX,t(8; 11)(p11; p15)	No therapy	Death after hospitalisation	Lee et al., 2008 [26]
AML	58, F	Not reported	Not reported	Not reported	Agerstam et al., 2007 [27]
AML	75, M	46,XY,t(8; 22) (p11; q11),t(9; 21)(q34; q22)	Not reported	Not reported	Fioretos et al., 2001 [28]
B-ALL	72, F	46,XX,t(8; 22),del(9)(p22),del(16)(q22),del(7)(p13)	1. R-Hyper-CVAD A and B + MTX 2. Flag-Ida	Originally, CR with MRD negative status; death 6 months after presentation	Barnes et al., 2020 [16]
B-ALL	48, M	46,XY,t(8; 22)(p11.2; q11.2)	1. Hyper-CVAD/MA 2. Allogeneic HSCT	Alive and disease-free at 12 months	Konishi et al., 2019 [29]
B-ALL	69, M	46,XY,t(8; 22)(p11.2; q11.2)	No therapy	Death after 3 months	Montenegro-Garreaud et al., 2017 [30]
B-ALL	58, F	45,XX,t(8; 22)(p11.2; q11.2),−16,add(19)(p13)	Allogeneic HSCT	Relapse with original clone; death after 13 months	Shimanuki et al., 2013 [31]
B-ALL	43, M	45,XY,t(6; 11)(q11; p13),−7,t(8; 22)(p11.2; q11.2),del(9) (p13p22)	Hyper-CVAD, hydroxyurea, and sorafenib (400 mg/d) and Flag-Ida Salvage	D15 BM after Flag-Ida: persistence of B-ALL and an expansion of myeloblasts, death 6 weeks later	Wakim et al., 2011 [19]
B-ALL	59, M	47,XY,t(8; 22)(p11.2; q11.2),+19[16]/46,XY[1]	Allogeneic HSCT	Alive and disease-free at 2 months	Kim et al., 2011 [32]
B-ALL (pre-B)	70, F	46,XX,t(8; 22)(p11; q11)[10]/45,idem,del(3)(p11p21),del(7)(p12p15),add(8)(p23),–9[6]/46,XX[4]	Vincristine, prednisone, daunorubicin, MTX, DEXA; MTX, 6-MP, vincristine, prednisone maintenance, intrathecal chemotherapy	Initially CR after induction therapy, progress after 8 months, death after 11 months from diagnosis	Baldazzi et al., 2010 [33]
B-ALL and myeloid/t-blasts in lymph node	50, M	46,XY,t(8; 22)(p11-12; q11)	Cord blood allogeneic HSCT	Alive and disease-free at 24 months	Morishige et al., 2013 [34]
B-ALL/MPN	41, F	46,XX,t(8; 22)(p11-12; q11)	Allogenic HSCT	Alive and disease-free at 15 months	Montenegro-Garreaud et al., 2017 [30]
B-ALL/MPN	56, F	47,XX,t(8; 22)(p11.2; q11.2),+der(22)t(8; 22)	Hyper-CVAD, ofatumumab, MTX, cytarabine, blinatumomab, ponatinib, hydroxyurea	Alive and disease-free at 13 months	Wang et al., 2016 [17]
B-ALL/MPN	21, M	46,XY,t(8; 22)(p12; q11)/45,idem,der(3; 9)(q10; q10),dic(7; 11)(p11; q13),+r(cp3)	UKALL 2003, Flag Salvage, Allogenic HSCT	Alive and disease-free at 24 months	Haslam et al., 2012 [35]
Trilineage mixed-phenotype acute leukemia	47, M	46,XY,t(8; 22)(p11-12; q11)	MEC, then ponatinib and hyper-CVAD (B), Allogeneic HSCT, Ponatinib maintenance	Alive and disease-free at 13.5 months	Khodadoust et al., 2015 [36]
CML	61, M	46,XY,t(8; 22)(p11.2; q11.2)	First imatinib, then ruxolitinib (concurrent JAK2 mutation), and then AZA + Ruxolitinib	No response on imatinib and ruxolitinib, normal WBC after adding AZA (follow-up 17 months)	Washburn et al., 2021 [15]
CML	41, M	46,XY,t(8; 22)(p11; q11)	Without treatment	Only followed up and monitored WBC count for 2 years, patient remained asymptomatic	Liu and Meng, 2018 [37]
CML	26, F	46,XX,t(8; 22)(p11; q11)	Hydroxyurea	Response to hydroxyurea; follow-up not reported	Qin et al., 2016 [38]
CML-AP	50, F	46,XX,t(8; 22)(p11.2; q11.2)[9]/46,t(8; 22)(p11.2; q11.2),i(9)(q10)[11]	Not reported	Death after 3 months in blast phase	Lee et al., 2008 [26]
CML-CP	28, M	46,XY,t(8; 22)(p11; q11)	Allogeneic HSCT	Alive and disease-free at 36 months	Chen et al., 2021 [39]
CML-CP	74, F	Not reported	Not reported	Not reported	Pini et al., 2002 [40]
CML-CP	65, F	Not reported	Hydroxyurea, IFNα	Not reported	Demiroglou et al., 2001 [41]
CML-CP	51, F	Not reported	Hydroxyurea, IFNα	Not reported	Demiroglou et al., 2001 [41]
CML-CP and B-cell proliferation	68, M	Not reported	Not available	Not reported	Murati et al., 2005 [42]
CML, evolved to blast crisis	55, F	46,XX,t(8; 22)(p11; q11)	Denied treatment	Not reported	Kapatia et al., 2021 [43]
CML, evolved to blast crisis	56, F	47,XX,t(3; 21)(q26; q22),t(8; 22)(p11; q11),+der(22)t(8; 22)	Hydroxyurea, IFNα, arsenic trioxide, cytarabine, daunorubicin	Death 24 months after presentation	Richebourg et al., 2008 [44]
CML/MPN	48, F	46,XX,t(8; 22)(p11.2; q11.2)	AZA and sorafenib	Asymptomatic, and continuing the treatment with azacitidine and sorafenib at 15 months follow-up	Dhangar et al., 2023 [14]
CML	8, M	46,XY,inv(4)(p15.2q13),t(8; 22; 14)(p11.2; q11.2; q24)	Allogeneic HSCT	Alive and disease-free at 60 months	Dolan et al., 2012 [45]
MPN	21, M	46,XY,t(8; 22)(p11-12; q11)	Allogeneic HSCT	Alive and disease-free at 48 months	Landberg et al., 2017 [46]
MPN	57, F	46,XX,t(8; 22)(p11.2; q11.2)	Allogeneic HSCT	Alive and disease-free at 42 months	Patnaik et al., 2010 [47]
MPN, evolved to T-LBL	53, M	46,XY,t(8; 22)(p11; q11)	1. Hydroxyurea 2. PETHEMA LAL-AR 2011 protocol	CR with negative MRD and complete metabolic response (PET–CT); follow-up not reported	Isaza et al., 2023 [48]

*Note:* AZA = azacitidine; CCyR = complete cytogenetic remission; Flag-Ida = fludarabine, idarubicin, cytarabine; Hyper-CVAD = cyclophosphamide, vincristine, doxorubicin alternating with methotrexate, cytarabine; IFNα = interferon *α*; PETHEMA LAL-AR 2011 protocol = vincristine, daunorubicin, prednisone, mitoxantrone, cytarabine, methotrexate, hydrocortisone, dexamethasone, L-asparaginase, mercaptopurine, cyclophosphamide, and teniposide; UKALL 2003 protocol = vincristine, daunorubicin, peg-asparaginase, cyclophosphamide, methotrexate, cytarabine, dexamethasone, and mercaptopurine; 7 + 3 = cytarabine, daunorubicin.

Abbreviations: ALL = acute lymphoblastic leukemia; AML = Acute myeloid leukemia; AP = accelerated phase; CML = chronic myeloid leukemia; CP = chronic phase; HSCT = hematopoietic stem cell transplantation; LBL = lymphoblastic lymphoma; MPN = myeloproliferative neoplasm; WBC = white blood count.

## Data Availability

The data that support the findings of this study are available on request from the corresponding authors. The data are not publicly available due to privacy or ethical restrictions.

## Supporting Information

PCR primers for MRD, Protocols for Cytogenetic and Fluorescence In Situ Hybridization (FISH) Analysis, Cytomorphological assessment, and FACS Analysis.

## References

[1] Gökbuget N. (2011). *Treatment Recommendation of the European Working Group for Adult ALL*.

[2] Iacobucci I., Mullighan C. G. (2017). Genetic Basis of Acute Lymphoblastic Leukemia. *Journal of Clinical Oncology*.

[3] Alaggio R., Amador C., Anagnostopoulos I. (2022). The 5th Edition of the World Health Organization Classification of Haematolymphoid Tumours: Lymphoid Neoplasms. *Leukemia*.

[4] Arber D. A., Orazi A., Hasserjian R. P. (2022). International Consensus Classification of Myeloid Neoplasms and Acute Leukemias: Integrating Morphologic, Clinical, and Genomic Data. *Blood*.

[5] Nowell P. C., Hungerford D. A. (1960). Chromosome Studies on Normal and Leukemic Human Leukocytes. *Journal of the National Cancer Institute*.

[6] Ravandi F. (2019). How I Treat Philadelphia Chromosome–Positive Acute Lymphoblastic Leukemia. *Blood*.

[7] Eswarakumar V. P., Lax I., Schlessinger J. (2005). Cellular Signaling by Fibroblast Growth Factor Receptors. *Cytokine & Growth Factor Reviews*.

[8] Kelleher F. C., O’Sullivan H., Smyth E., McDermott R., Viterbo A. (2013). Fibroblast Growth Factor Receptors, Developmental Corruption and Malignant Disease. *Carcinogenesis*.

[9] Chen B., Liu S., Gan L. (2018). FGFR1 Signaling Potentiates Tumor Growth and Predicts Poor Prognosis in Esophageal Squamous Cell Carcinoma Patients. *Cancer Biology & Therapy*.

[10] Du S., Zhang Y., Xu J. (2023). Current Progress in Cancer Treatment by Targeting FGFR Signaling. *Cancer Biol Med*.

[11] Raess P. W. (2018). Myelodysplastic/Myeloproliferative Neoplasms. *The Atlas of Genetics and Cytogenetics in Oncology and Haematology*.

[12] Gotlib J., Kiladjian J. J., Vannucchi A. (2021). A Phase 2 Study of Pemigatinib (FIGHT-203; INCB054828) in Patients With Myeloid/Lymphoid Neoplasms (MLNs) With Fibroblast Growth Factor Receptor 1 (FGFR1) Rearrangement (MLN *FGFR1*). *Blood*.

[13] Zhang Z., Zhu Y., Wang Z. (2023). Case Report: A Novel FGFR1 Fusion in Acute B-lymphoblastic Leukemia Identified by RNA Sequencing. *Frontiers Oncology*.

[14] Dhangar S., Shanmukhaiah C., Sawant L. (2023). Synergetic Effect of Azacitidine and Sorafenib in Treatment of a Case of Myeloid Neoplasm With Sole Chromosomal Abnormality t(8;22)(p11.2;q11.2)/BCR-FGFR1 Rearrangement. *Cancer Genetics*.

[15] Washburn E., Bayerl M. G., Ketterling R. P., Malysz J. (2021). A Rare Case of Atypical Chronic Myeloid Leukemia Associated With t(8;22)(p11.2;q11.2)/BCR-FGFR1 Rearrangement: A Case Report and Literature Review. *Cancer Genetics*.

[16] Barnes E. J., Leonard J., Medeiros B. C., Druker B. J., Tognon C. E. (2020). Functional Characterization of Two Rare BCR–FGFR1^+^ Leukemias. *Molecular Case Studies*.

[17] Wang W., Tang G., Kadia T. (2016). Cytogenetic Evolution Associated With Disease Progression in Hematopoietic Neoplasms With t(8;22)(p11;q11)/*BCR-FGFR1* Rearrangement. *Journal of the National Comprehensive Cancer Network*.

[18] Matikas A., Tzannou I., Oikonomopoulou D., Bakiri M. (2013). A Case of Acute Myelogenous Leukaemia Characterised by the BCR-FGFR1 Translocation. *BMJ Case Reports*.

[19] Wakim J. J., Tirado C. A., Chen W., Collins R. (2011). t(8;22)/BCR-FGFR1 Myeloproliferative Disorder Presenting as B-acute Lymphoblastic Leukemia: Report of a Case Treated With Sorafenib and Review of the Literature. *Leukemia Research*.

[20] Hiller B., Bradtke J., Balz H., Rieder H. (2004). CyDAS Online Analysis Site. http://www.cydas.org/OnlineAnalysis/.

[21] Goekbuget N., Viardot A., Steffen B. (2022). Outcome of 841 Older Patients (> 55 Years) With Newly Diagnosed Ph/BCR-ABL Negative ALL Prospectively Treated According to Pediatric-Based, Age-Adapted GMALL Protocols. *Blood*.

[22] Goekbuget N., Stelljes M., Viardot A. (2021). First Results of the Risk-Adapted, MRD-Stratified GMALL Trial 08/2013 in 705 Adults With Newly Diagnosed Acute Lymphoblastic Leukemia/Lymphoma (ALL/LBL). *Blood*.

[23] Gökbuget N., Kneba M., Raff T. (2012). Adult Patients With Acute Lymphoblastic Leukemia and Molecular Failure Display a Poor Prognosis and are Candidates for Stem Cell Transplantation and Targeted Therapies. *Blood*.

[24] Jackson C. C., Medeiros L. J., Miranda R. N. (2010). 8p11 Myeloproliferative Syndrome: A Review. *Human Pathology*.

[25] Li T., Zhang G., Zhang X., Lin H., Liu Q. (2022). The 8p11 Myeloproliferative Syndrome: Genotypic and Phenotypic Classification and Targeted Therapy. *Frontiers Oncology*.

[26] Lee S. G., Park T. S., Lee S. T. (2008). Rare Translocations Involving Chromosome Band 8p11 in Myeloid Neoplasms. *Cancer Genetics and Cytogenetics*.

[27] Ågerstam H., Lilljebjörn H., Lassen C. (2007). Fusion Gene-Mediated Truncation ofRUNX1 as a Potential Mechanism Underlying Disease Progression in the 8p11 Myeloproliferative Syndrome. *Genes, Chromosomes and Cancer*.

[28] Fioretos T., Panagopoulos I., Lassen C. (2001). Fusion of the *BCR* and the Fibroblast Growth Factor Receptor-1 (*FGFR1*) Genes as a Result of t(8;22)(p11;q11) in a Myeloproliferative Disorder: The First Fusion Gene Involving *BCR* but not *ABL*: Fusion of the *BCR* and *FGFR1* Genes. *Genes, Chromosomes and Cancer*.

[29] Konishi Y., Kondo T., Nakao K. (2019). Allogeneic Hematopoietic Stem Cell Transplantation for 8p11 Myeloproliferative Syndrome With BCR-FGFR1 Gene Rearrangement: A Case Report and Literature Review. *Bone Marrow Transplantation*.

[30] Montenegro-Garreaud X., Miranda R. N., Reynolds A. (2017). Myeloproliferative Neoplasms With t(8;22)(p11.2;q11.2)/BCR-FGFR1: A Meta-Analysis of 20 Cases Shows Cytogenetic Progression With B-Lymphoid Blast Phase. *Human Pathology*.

[31] Shimanuki M., Sonoki T., Hosoi H. (2013). Acute Leukemia Showing t(8;22)(p11;q11), Myelodysplasia, CD13/CD33/CD19 Expression and Immunoglobulin Heavy Chain Gene Rearrangement. *Acta Haematologica*.

[32] Kim S. Y., Oh B., She C. J. (2011). 8p11 Myeloproliferative Syndrome With BCR-FGFR1 Rearrangement Presenting With T-lymphoblastic Lymphoma and Bone Marrow Stromal Cell Proliferation: A Case Report and Review of the Literature. *Leukemia Research*.

[33] Baldazzi C., Iacobucci I., Luatti S. (2010). B-Cell Acute Lymphoblastic Leukemia as Evolution of a 8p11 Myeloproliferative Syndrome With t(8;22)(p11;q11) and BCR-FGFR1 Fusion Gene. *Leukemia Research*.

[34] Morishige S., Oku E., Takata Y. (2013). A Case of 8p11 Myeloproliferative Syndrome with BCR-FGFR1 Gene Fusion Presenting With Trilineage Acute Leukemia/Lymphoma, Successfully Treated by Cord Blood Transplantation. *Acta Haematologica*.

[35] Haslam K., Langabeer S. E., Kelly J., Coen N., O’Connell N. M., Conneally E. (2012). Allogeneic Hematopoietic Stem Cell Transplantation for a BCR-FGFR1 Myeloproliferative Neoplasm Presenting as Acute Lymphoblastic Leukemia. *Case Reports in Hematology*.

[36] Khodadoust M. S., Luo B., Medeiros B. C. (2016). Clinical Activity of Ponatinib in a Patient With FGFR1-Rearranged Mixed-Phenotype Acute Leukemia. *Leukemia*.

[37] Liu J., Meng L. (2018). 8p11 Myeloproliferative Syndrome With t(8;22)(p11;q11): A Case Report. *Experimental and Therapeutic Medicine*.

[38] Qin Y. W., Yang Y. N., Bai P., Wang C. (2016). Chronic Myelogenous Leukemia-Like Hematological Malignancy With t(8;22) in a 26-Year-Old Pregnant Woman: A Case Report. *Oncology Letters*.

[39] Chen X., Huang L., Zheng C., Wang Z. (2021). A Case of a Patient Characterized by t(8;22)(p11;q11) and BCR/FGFR1 Fusion Gene, Who was Successfully Treated With Haploidentical Hematopoietic Stem Cell Transplantation. *Hematology*.

[40] Pini M., Gottardi E., Scaravaglio P. (2002). A Fourth Case of BCR-FGFR1 Positive CML-Like Disease With t(8;22) Translocation Showing an Extensive Deletion on the Derivative Chromosome 8p. *The Hematology Journal*.

[41] Demiroglu A., Steer E. J., Heath C. (2001). The t(8;22) in Chronic Myeloid Leukemia Fuses BCR to FGFR1: Transforming Activity and Specific Inhibition of FGFR1 Fusion Proteins. *Blood*.

[42] Murati A., Arnoulet C., Lafage-Pochitaloff M. (2005). Dual Lympho-Myeloproliferative Disorder in a Patient with t(8;22) With BCR-FGFR1 Gene Fusion. *International Journal of Oncology*.

[43] Kapatia G., Remani A. S. N., Naseem S., Parihar M., Sreedharanunni S. (2021). Myeloid Neoplasm With t(8;22)(p11;q11): A Mimicker of Chronic Myeloid Leukaemia in Blast Crisis. *Indian Journal of Hematology and Blood Transfusion*.

[44] Richebourg S., Theisen O., Plantier I. (2008). Chronic Myeloproliferative Disorder with t(8;22)(p11;q11) can Mime Clonal Cytogenetic Evolution of Authentic Chronic Myelogeneous Leukemia. *Genes, Chromosomes and Cancer*.

[45] Dolan M., Cioc A., Cross N. C. P., Neglia J. P., Tolar J. (2012). Favorable Outcome of Allogeneic Hematopoietic Cell Transplantation for 8p11 Myeloproliferative Syndrome Associated With BCR-FGFR1 Gene Fusion: BMT for Atypical 8p11 Syndrome. *Pediatric Blood and Cancer*.

[46] Landberg N., Dreimane A., Rissler M., Billström R., Ågerstam H. (2017). Primary Cells in *BCR/FGFR1*‐Positive 8p11 Myeloproliferative Syndrome are Sensitive to Dovitinib, Ponatinib, and Dasatinib. *European Journal of Haematology*.

[47] Patnaik M. M., Gangat N., Knudson R. A. (2010). Chromosome 8p11.2 Translocations: Prevalence, FISH Analysis for *FGFR1* and *MYST3*, and Clinicopathologic Correlates in a Consecutive Cohort of 13 Cases From a Single Institution. *American Journal of Hematology*.

[48] Isaza A. P., Quintero S. C., González L. P. Q., Córdoba F. E. A., Olivar A. F. A., Ocaña J. C. B. (2023). Myeloid/Lymphoid Neoplasm With Eosinophilia and BCR/FGFR1 Rearrangement With Transformation to Cortical T-lymphoblastic Lymphoma and Erythroid Precursors: A Case Report. *Journal of Medical Case Reports*.

[49] Pattarozzi A., Carra E., Favoni R. E. (2017). The Inhibition of FGF Receptor 1 Activity Mediates Sorafenib Antiproliferative Effects in Human Malignant Pleural Mesothelioma Tumor-Initiating Cells. *Stem Cell Research & Therapy*.

[50] Gozgit J. M., Wong M. J., Moran L. (2012). Ponatinib (AP24534), A Multitargeted Pan-FGFR Inhibitor With Activity in Multiple FGFR-Amplified or Mutated Cancer Models. *Molecular Cancer Therapeutics*.

[51] Chase A., Grand F. H., Cross N. C. P. (2007). Activity of TKI258 Against Primary Cells and Cell Lines With FGFR1 Fusion Genes Associated With the 8p11 Myeloproliferative Syndrome. *Blood*.

[52] Chase A., Bryant C., Score J., Cross N. C. P. (2013). Ponatinib as Targeted Therapy for FGFR1 Fusions Associated With the 8p11 Myeloproliferative Syndrome. *Haematologica*.

[53] Abou-Alfa G. K., Sahai V., Hollebecque A. (2021). Pemigatinib for Previously Treated Locally Advanced/Metastatic Cholangiocarcinoma (CCA): Update of Fight-202. *Journal of Clinical Oncology*.

[54] Wu L., Zhang C., He C. (2021). Discovery of Pemigatinib: A Potent and Selective Fibroblast Growth Factor Receptor (FGFR) Inhibitor. *Journal of Medicinal Chemistry*.

[55] Pace A., Scirocchi F., Napoletano C. (2023). Targeting FGFRS by Pemigatinib Induces G1 Phase Cell Cycle Arrest, Cellular Stress and Upregulation of Tumor Suppressor Micrornas. *Journal of Translational Medicine*.

