# Supplementary material for: Acute Lymphoblastic Leukemia Characterized by Rare BCR::FGFR1 Translocation: A Case Report With Literature Review
Source: Case Rep Hematol. 2025 Oct 23;2025:8892036. doi: 10.1155/crh/8892036 (PMC12575048; doi:10.1155/crh/8892036)
Supplement: Supporting Information — Additional supporting information can be found online in the Supporting Information section. [file 8892036.f1.docx]

**Supplementary Material**

**For minimal residual disease measurement, the following PCR primers were used:**

**Gene**: IGH DJ

**D-J usage**: D2-2 - J6

**Rearrangement**: D2-2 (-6/7/0) J6

**Forward Primer**: GTAGTACCAGCTGCCCTTCTTA

**Reverse Primer**: GCAGAAAACAAAGGCCCTAGAGT

**Probe**: FAM-ACCACGGTCACCGTCTCCTCAGGTAAG-TAM

**Complete Sequence**: CATAGTATCAGCAGGAGAACTAGCCAGAGACAGCAAGAggggactcagtgactcccgcggggacaggaggattttgtgggggctcgtgtcactgtgaggatattgtagtagtaccagctgc_CCTTCTT_attactactactactacggtatggacgtctggggccaagggaccacggtcaccgtctcctcaGGTAAGAATGGCCACTCTAGGGCCTTTGTT

**Cytogenetic and Fluorescence In Situ Hybridization (FISH) Analysis:**Bone marrow cells were cultured and cytogenetically analyzed using standard procedures. The karyotype was documented according to the 2020 International System for Human Cytogenomic Nomenclature (ISCN).
FISH analysis was performed on metaphase cells (sample prepared after 24 hours of bone marrow cell cultivation without supplements) following standard protocols, utilizing the following commercially available probes: XL FGFR1 Break Apart Probe by MetaSystems (D-5041-100-OG), XL CDKN2A/9q22 Deletion Probe by MetaSystems (D-5118-100-OG), and Vysis LSI BCR/ABL Dual Color, Dual Fusion Translocation Probe Kit by Abbott (08L10-001) in combination with Vectashield Antifade Mounting Medium with DAPI (Vector Laboratories).

**Cytomorphological assessment:**

For cytomorphological assessment, bone marrow samples were collected in EDTA tubes and applied to glass slides. Bone marrow fragments were then gathered using a glass slide or pipette and transferred to another slide. A second slide was placed over the sample, and the slides were gently pressed and moved against each other to ensure an even distribution of the material. The prepared slides were air-dried for at least 30 minutes.

For staining, slides were placed in staining cuvettes and processed according to the Pappenheim staining protocol. This involved staining with May-Grünwald solution for 5 minutes, followed by rinsing in a buffer solution, staining with Giemsa solution (diluted 1:10) for 30 minutes, a second rinse in buffer, and a final rinse with distilled water. After air-drying, the slides were labelled for microscopy.

Microscopic evaluation entailed screening a minimum of four smears, adjusted to the clinical context. The entire smear was scanned in a meandering pattern at varying magnifications. Initially, a 10x magnification objective (without oil) was used to assess the overall cell content, megakaryopoiesis, and any abnormal cells. Subsequently, specific lineages were examined under oil immersion (40x, 63x, or 100x) to evaluate cell proportions and morphology, with cell counts typically ranging from 200 to 500 cells. The reagents used included May-Grünwald stain (Merck), buffer tablets (pH 6.8, Merck), and Giemsa stain (Merck, diluted 1:10 with buffer).

**FACS Analysis:**

Samples for immunophenotypic analysis were collected in EDTA tubes. Immunophenotyping was performed as outlined by Ludwig et al. (1998) (**REF**), using a Navios flow cytometer (Beckman Coulter, Krefeld, Germany) and analysed with Kaluza software. Commercially available fluorochrome-conjugated monoclonal antibodies (MoAbs) specific to B-, T-, myeloid-, and progenitor-cell antigens were employed in dual-staining experiments. A standardised panel of monoclonal antibodies was applied to assess bone marrow samples, with surface antigen expression deemed positive when 20% or more of leukemic cells exhibited fluorescence intensity above the negative control. For cytoplasmic antigens, positivity was defined by a threshold of ≥10% leukemic cells. In all experiments, isotype-matched non-reactive mouse MoAbs at equivalent protein concentrations were used as negative controls. Surface antigen fluorescence was immediately evaluated by flow cytometry post-immunostaining.

The FACS analysis targeted markers such as CD10, CD19, CD34, and HLA-DR, essential for identifying common B-ALL. Immunophenotypic subtyping adhered to the EGIL criteria for B-cell precursor ALL, distinguishing subtypes including pro-B-ALL, common ALL, pre-B-ALL, and mature B-ALL.

(**REF**) Wolf-Dieter Ludwig, Harald Rieder, Claus R. Bartram, Barbara Heinze, Stefan Schwartz, Winfried Gassmann, Helmut Löffler, Dieter Hossfeld, Gerhard Heil, Susanne Handt, Axel Heyll, Helmut Diedrich, Konstanze Fischer, Adelheid Weiss, Bernd Völkers, Ülker Aydemir, Christa Fonatsch, Nicola Gökbuget, Eckhard Thiel, Dieter Hoelzer,
*Immunophenotypic and Genotypic Features, Clinical Characteristics, and Treatment Outcome of Adult Pro-B Acute Lymphoblastic Leukemia: Results of the German Multicenter Trials GMALL 03/87 and 04/89*, Blood, Volume 92, Issue 6, 1998, Pages 1898–1909, ISSN 0006-4971, <https://www.sciencedirect.com/science/article/pii/S0006497120740589>.
